# City Hospital

**Published:** 1859-07

**Authors:** 


### CITY HOSPITAL.

We are happy to inform the readers of the *Journal*, that the City Hospital is now completed, and arrangements have been made by the municipal authorities for opening the same, for the reception of patients. The site of the building, in the Southern portion of the city, was selected most judiciously for the salubrity of its location. It has been constructed upon the most approved plans, for perfect ventilation and convenience, and will admirably fulfil the purposes for which it was established. It is capable of accommodating comfortably about two hundred patients, and will be under the charge of the following Board of Physicians and Surgeons.

*Physicians*—DeLaskie Miller, M.D., Joseph K. Ross, M.D., and Samuel C. Blake, M.D.

*Surgeons*—Daniel Brainard, M.D., Geo. K. Amerman, M.D., and Geo. Schloetzer, M.D.

A ward will be appropriated for the reception and treatment of diseases of the eye, which will be attended by E. L. Holmes, M.D., one of the attending surgeons of the Chicago Charitable Eye and Ear Infirmary.

A lying-in ward for parturient women will also be connected with the Hospital.

Thus we may affirm that the city, for the first time, possesses an institution for the care of the sick, in all respects, commensurate with the demands of the public and interests of the profession.

This arrangement affords ample facilities for clinical instruction, which will be given in all the departments, to those pursuing their medical studies in this city.

We feel confident the profession will appreciate the great advantages thus afforded for the attainment of a thorough knowledge of clinical medicine.

The rate of charges in the public wards for all patients, either from this city, or from a distance, will be $3,50 per week, which will embrace medical and surgical attendance, medicines, board, lodging and nursing. For those who wish private rooms and special attendance, such rates as may be fixed, on the application of the patient. M.

